# Mosquito vector proteins homologous to α1-3 galactosyl transferases of tick vectors in the context of protective immunity against malaria and hypersensitivity to vector bites

**DOI:** 10.1186/s13071-021-04801-7

**Published:** 2021-06-05

**Authors:** Ranjan Ramasamy

**Affiliations:** ID-FISH Technology, 556 Gibraltar Drive, Milpitas, CA95035 USA

**Keywords:** α-Gal, α-Gal syndrome, α1-3 Galactosyl epitopes, Antibodies to α-gal, Galactosyl transferases, Hypersensitivity, Immune protection, Malaria, Mosquito vectors, Tick vectors

## Abstract

**Background:**

An epitope, Galα1-3Galβ1-4GlcNAc-R, termed α-gal, is present in glycoconjugates of New World monkeys (platyrrhines) and other mammals but not in hominoids and Old World monkeys (catarrhines). The difference is due to the inactivation of α1-3 galactosyl transferase (α1-3 GT) genes in catarrhines. Natural antibodies to α-gal are therefore developed in catarrhines but not platyrrhines and other mammals. Hypersensitivity reactions are commonly elicited by mosquito and tick vector bites. IgE antibodies against α-gal cause food allergy to red meat in persons who have been exposed to tick bites. Three enzymes synthesising the terminal α1-3-linked galactose in α-gal, that are homologous to mammalian α and β1-4 GTs but not mammalian α1-3 GTs, were recently identified in the tick vector *Ixodes scapularis*. IgG and IgM antibodies to α-gal are reported to protect against malaria because mosquito-derived sporozoites of malaria parasites express α-gal on their surface. This article explores the possibility that the α-gal in sporozoites are acquired from glycoconjugates synthesised by mosquitoes rather than through de novo synthesis by sporozoites.

**Methods:**

The presence of proteins homologous to the three identified tick α1-3 GTs and mammalian α1-3 GTs in two important mosquito vectors, *Aedes aegypti* and *Anopheles gambiae*, as well as *Plasmodium* malaria parasites, was investigated by BLASTp analysis to help clarify the source of the α-gal on sporozoite surfaces.

**Results:**

*Anopheles gambiae* and *Ae. aegypti* possessed several different proteins homologous to the three *I. scapularis* proteins with α1-3 GT activity, but not mammalian α1-3 GTs. The putative mosquito α1-3 GTs possessed conserved protein domains characteristic of glycosyl transferases. However, the genus *Plasmodium* lacked proteins homologous to the three *I. scapularis* proteins with α1-3 GT activity and mammalian α1-3 GTs.

**Conclusions:**

The putative α1-3 GTs identified in the two mosquito vectors may synthesise glycoconjugates containing α-gal that can be transferred to sporozoite surfaces before they are inoculated into skin during blood feeding. The findings merit further investigation because of their implications for immunity against malaria, hypersensitivity to mosquito bites, primate evolution, and proposals for immunisation against α-gal.

**Graphic abstract:**

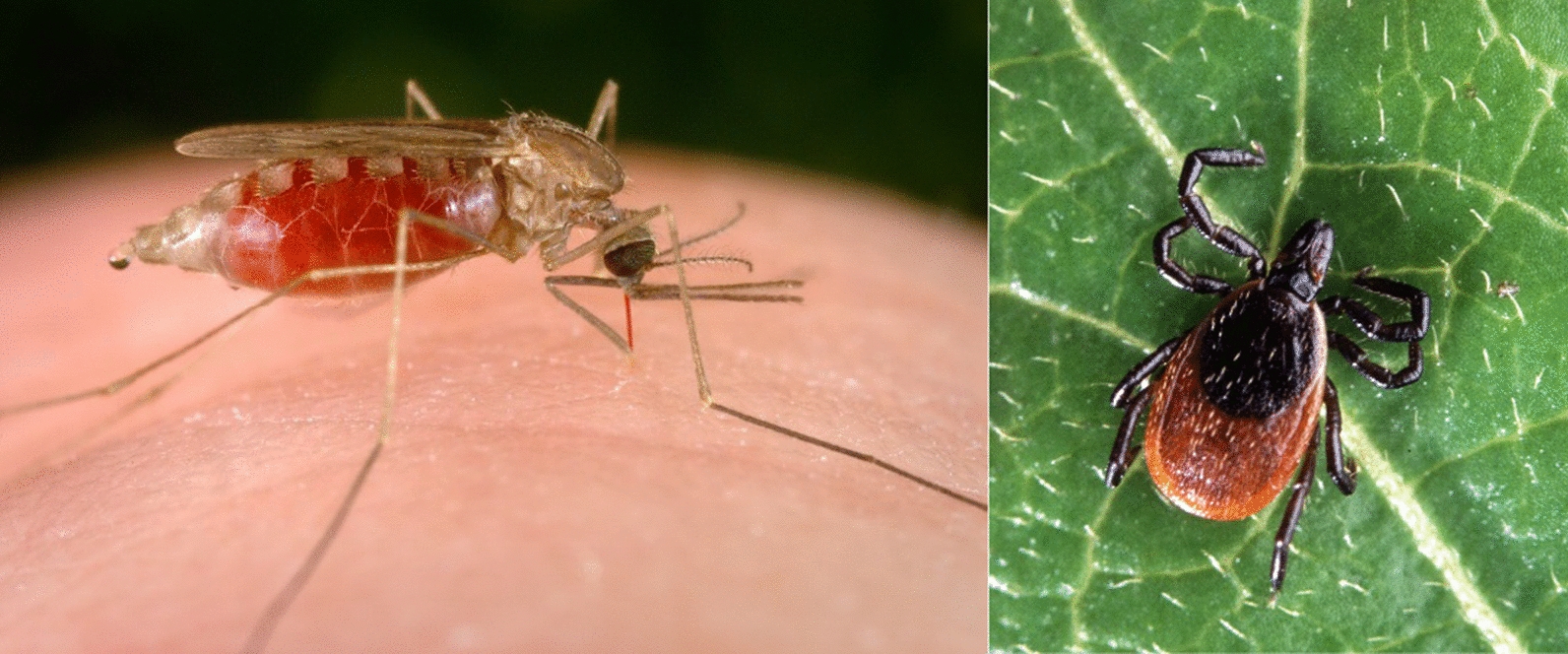

**Supplementary Information:**

The online version contains supplementary material available at 10.1186/s13071-021-04801-7.

Infected female vector mosquitoes taking a human blood meal transmit parasites (e.g. *Plasmodium* malaria parasites) and arboviruses (e.g. dengue virus) that cause serious diseases of global concern. Mosquitoes inject saliva into the skin to facilitate blood feeding. Components of mosquito saliva, that include many proteins, can cause immediate and delayed hypersensitivity reactions involving IgE and IgG antibodies as well as T lymphocytes in the skin at the bite site, resulting sometimes in severe delocalised and systemic pathology [1]. Ticks are also arthropod vectors that transmit parasites (e.g. *Babesia* species), bacteria (e.g. *Borrelia* species), and viruses (e.g. tick-borne encephalitis virus) acquired from wild animals to cause globally prevalent human diseases [2]. Ticks are rare opportunistic feeders on humans, and therefore, less is known about hypersensitivity reactions to tick salivary components injected into humans during blood feeding. It is, however, known that immunisation of guinea pigs with tick saliva glycoproteins produces immune-mediated rejection of ticks feeding on guinea pigs [3].

A type of food allergy to mammalian meat is now recognised as a type 1 hypersensitivity reaction that occurs in a proportion of people who have been exposed to tick bites [4]. IgE antibodies to the trisaccharide epitope Galα1-3Galβ1-4GlcNAc-R (α-gal), initially elicited by molecules in tick saliva containing α-gal, bind to α-gal from red meat and cause the hypersensitivity, which has been termed the α-gal syndrome (AGS) [4]. AGS can produce clinical manifestations ranging from urticaria to fatal anaphylaxis [4]. Human serum, however, contains natural antibodies of IgG, IgM and IgA isotypes to α-gal that constitute approximately 1% of all immunoglobulins in serum, and these antibodies are elicited by α-gal-containing glycolipids present in commensal gut bacteria [5–8]. Humans, apes and Old World monkeys (termed catarrhines) lack α-gal and are therefore able to the produce antibodies to α-gal, while new world monkeys (termed platyrrhines), lemurs and other mammals possess α-gal and, because of self-tolerance, are unable to produce antibodies to α-gal [5–8]. Inactivating mutations in α1-3 galactosyl transferases (α1-3 GTs), estimated to have occurred 20–28 million years ago as two separate events in Old World monkeys and hominids during catarrhine evolution, are responsible for this difference [9].

The α-gal in the tick vector *Ixodes scapularis* has been shown to be synthesised by two tick enzymes that are homologous to an α1-4 GT in humans responsible for synthesising globosides (also termed Gb3 synthase), and a third enzyme which is homologous to a human β1-4 GT [10]. The α1-3 GT activity of the three *I. scapularis* enzymes has been attributed to an altered specificity that arose during evolution [10], which may have been facilitated by an expansion of the numbers of α and β GTs in *I. scapularis* [10]. The presence of α-gal in the salivary glands of *Anopheles* mosquito vectors and in *Plasmodium* sporozoites obtained from *Anopheles* salivary glands has been reported, but whether the α-gal found on sporozoites was synthesised by sporozoites or acquired from the *Anopheles* vector was not established [11]. Because of important implications for protective immunity against mosquito-borne pathogens, hypersensitivity due to mosquito bites, and primate evolution, the possible presence of α1-3 GTs in the principal African malaria vector *Anopheles gambiae*, the primary global arboviral vector *Aedes aegypti* and the genus *Plasmodium* was investigated by in silico protein sequence homology analysis.

The existence of proteins with homology to the three identified tick α1-3 GTs [10] and three murine α1-3 GTs was explored by NCBI BLASTp searches (https://blast.ncbi.nlm.nih.gov/Blast.cgi?PAGE=Proteins) against all non-redundant protein sequences of *An. gambiae* (NCBI taxid 7165), *Ae. aegypti* (NCBI taxid 7159) and the genus *Plasmodium* (NCBI taxid 5820). These protein sequences available at NCBI are mostly predicted from gene sequences obtained by whole genome sequencing. Search parameters utilised in the BLASTp analysis were as follows: matrix—BLOSUM62; gap costs—existence 11, extension 1; alignment initiation length 6; conditional compositional score matrix adjustment; threshold *E* value 0.05. The three *I. scapularis* protein sequences used as queries in BLASTp were obtained from the UniProtKB/Swiss-Prot database (https://www.uniprot.org) using the VectorBase *I. scapularis* gene identities from reference 10. Two of the three *I. scapularis* enzymes with α1-3 GT activity are annotated in the UniProtKB/Swiss-Prot protein database as α1-4 *N*-acetylglucosaminyl transferases (B7QKR3 and BFPLD1) and the third as a xylosylprotein β galactosyl transferase (B7PFJ6).

Protein sequences from the three known functional mammalian α1-3 GTs from *Mus musculus* were also used in BLASTp analysis against non-redundant protein sequences of *An. gambiae* (NCBI taxid 7165), *Ae. aegypti* (NCBI taxid 7159) and the genus *Plasmodium* (NCBI taxid 5820) using the BLASTp parameters described above. The murine α1-3 GT protein sequences used were as follows: (i) glycoprotein α1-3 galactosyl transferase with NCBI reference NP_001139293.1; (ii) cis AB transferase with NCBI reference BAB20560.1; (iii) isoglobotriasoylceramide synthase from UniProtKB/Swiss-Prot with the reference Q3VIN9. Additional IDs for mosquito proteins identified through BLASTp were obtained from VectorBase (https://vectorbase.org/vectorbase/app).

Conserved functional domains in homologous proteins and phylogenetic trees for them in the NCBI Tree viewer using the nearest-neighbour-joining tree method and Kimura distance option were determined through the BLASTp output search options.

Proteins homologous to the three *I. scapularis* enzymes were not detected in the genus *Plasmodium* by BLASTp analysis at *E* ≤ 0.05. Additionally, BLASTp analysis of the genus *Plasmodium*, *An. gambiae* and *Ae. aegypti* with the three functional mammalian α1-3 GTs did not identify homologous proteins at *E* ≤ 0.05. However, both *An. gambiae* and *Ae. aegypti* possessed several unique proteins that were homologous to each of the three *I. scapularis* enzymes with α1-3 GT activity, with *E* values that were much less than 0.05, showing that all observed sequence homologies were highly significant (Table 1). The high alignment scores and query cover showed that the homologies between the tick and mosquito proteins extended over most of the length of each pair of proteins that were compared. Furthermore, no other unique homologous *Aedes* or *Anopheles* proteins other than those listed in Table 1 were identified by BLASTp analysis against the three *I. scapularis* GTs. The two enzymes with α1-3 GT activity but annotated as α1-4 *N*-acetylglucosaminyl transferases from *I. scapularis* identified a set of three *Ae. aegypti* proteins, with each of the three proteins having different E values and percent identities against the two *I. scapularis* enzymes. These two *I. scapularis* enzymes also identified a set of four *An. gambiae* proteins with each of the four proteins having different E values and percent identities against the two *I. scapularis* enzymes. This suggests that the three putative *Ae. aegypti* GTs identified in this way have close sequence homology with each other as do the four putative *An. gambiae* GTs with each other, and also that the two *I. scapularis* α1-3 GTs share sequence homology. Sequence homology also exists within the three putative *Ae. aegypti* GTs and within the two putative *An. gambiae* GTs that were identified using the single *I. scapularis* protein annotated as a xylosylprotein β galactosyl transferase but possessing α1-3 GT activity. These homologies are further illustrated in the phylogenetic trees constructed for each of the three *I. scapularis* enzymes and their homologous *Ae. aegypti* and *An. gambiae* proteins (Additional file 1).

**Table 1:** *Aedes aegypti* and *Anopheles gambiae* homologues of *Ixodes scapularis* enzymes with α1-3 galactosyl transferase activity identified by NCBI BLASTp analyses

NCBI accession ID (VectorBase protein ID)	*E* value	Percent identity	Query cover (%)	Max. score	Total score	Accession length
*Ae. aegypti* homologues of *I. scapularis* B7QKR3 (ISCW024908) annotated as α1-4 *N*-acetylglucosaminyl transferase (length 300 amino acids)						
XP_001654206.1* (AAEL001900-PA)	3.00E−25	28.47	90	103	103	371
XP_001650194.1 (AAEL005019-PA)	1.00E−18	24.18	88	85.5	85.5	354
XP_001654207.2 (AAEL001895-PA)	2.00E−18	23.55	88	85.1	85.1	404
*Ae. aegypti* homologues of *I. scapularis* B7PLD1 (ISCW006262) annotated as α1-4 *N*-acetylglucosaminyl transferase (length 344 amino acids)						
XP_001650194.1 (AAEL005019-PA)	2.00E−41	32.86	79	148	148	354
XP_001654206.1* (AAEL001900-PA)	6.00E−34	27.11	78	129	129	371
XP_001654207.2 (AAEL001895-PA)	1.00E−24	25.75	80	103	103	404
*Ae. aegypti* homologues of *I. scapularis* B7PFJ6 (ISCW003979) annotated as xylosylprotein β galactosyl transferase (length 290 amino acids)						
XP_001653000.2* (AAEL007895-PA)	6.00E−102	48.79	98	301	301	313
XP_021707280.1 (AAEL019794-PA)	8.00E−32	40.91	52	123	123	485
XP_021703090.1 (AAEL021029)	2.00E−29	37.91	51	114	114	318
*An. gambiae* homologues of *I. scapularis* B7QKR3 (ISCW024908) annotated as α1-4 *N*-acetylglucosaminyl transferase (length 300 amino acids)						
XP_555205.1* (AGAP008260-PA)	8.00E−23	25.46	88	96.7	96.7	404
XP_317211.3 (AGAP008258-PA)	3.00E−22	25.27	88	94.7	94.7	358
XP_001237894.2 (AGAP008259-PA)	5.00E−22	26.74	88	93.2	93.2	302
XP_555204.2 (AGAP008261-PA)	1.00E−07	22.74	90	52	52	347
*An. gambiae* homologues of *I. scapularis* B7PLD1 (ISCW006262) annotated as α1-4 *N*-acetylglucosaminyl transferase (length 344 amino acids)						
XP_001237894.2 (AGAP008259-PA)	4.00E−49	33.22	83	166	166	302
XP_317211.3 (AGAP008258-PA)	3.00E−44	31.79	83	155	155	358
XP_555205.1* (AGAP008260-PA)	2.00E−41	31.58	84	149	149	404
XP_555204.2 (AGAP008261-PA)	5.00E−17	27.46	78	80.1	80.1	347
*An. gambiae* homologues of *I. scapularis* B7PFJ6 (ISCW003979) annotated as xylosylprotein β galactosyl transferase (length 290 amino acids)						
XP_318033.4 (AGAP0048781-PA)	1.00E−30	31.28	72	116	116	308
XP_001689009.1 (AGAP008285-PA)	1.00E−27	32.86	71	108	108	322

Data are the results of BLASTp searches for different mosquito proteins with significant homology (*E* ≤ 0.05) to the three *Ixodes scapularis* enzymes. Asterisks indicate proteins that were also identified in reference 10. The UniProtKB/Swiss-Prot IDs with their VectorBase gene IDs in parentheses are shown for *I. scapularis* proteins. Homologous mosquito proteins are shown with their NCBI accession IDs with VectorBase IDs in parentheses

The two *I. scapularis* enzymes annotated as α1-4 *N*-acetylglucosaminyl transferases but possessing α1-3 GT activity, and all their homologous putative *Ae. aegypti* and *An. gambiae* GTs shown in Table 1 possessed two conserved protein domains: (i) pfam 04572 characteristic of globoside synthases; and (ii) cl 19952 which contains a UDP-galactose binding region with a DXD amino acid motif. The *I. scapularis* protein annotated as a xylosylprotein β galactosyl transferase but with α1-3 GT activity and all the identified homologous putative *Ae. aegypti* and *An. gambiae* α1-3 GTs shared the conserved protein domain cl 11394 which is representative of glycosyl transferase family A with a GT-A type of structural fold, as well as the DXD amino acid motif which participates in binding divalent metal ions essential for GT activity [12].

Based on the binding of B4 lectin from *Bandeiraea (Griffonia) simplicifolia* which specifically recognises terminal α-galactosyl residues and a monoclonal antibody specific for the α-gal epitope, as well as abrogation of their binding by prior α-galactosidase treatment, α-gal was reported to be present in the salivary glands of *Anopheles* vectors as well as *Plasmodium falciparum* and *Plasmodium berghei* sporozoites obtained from *Anopheles* salivary glands [11]. Investigations of the protection conferred by antibodies to α-gal against infection with the murine malaria parasite *P. berghei* in genetically engineered mice unable to synthesise α-gal were then undertaken [11]. Antibodies to α-gal in such mice conferred partial protection against blood-stage infection by *P. berghei* sporozoites inoculated by mosquito bites, but not intravenously injected *P. berghei* sporozoites [11]. The study produced evidence supporting roles for IgM and some IgG isotypes of antibodies to α-gal, complement and phagocytic cells in immune damage to *P. berghei* sporozoites at the skin inoculation site [11]. However, it was not able to definitively differentiate between an origin for the α-gal-containing glycoconjugates in anopheline salivary glands that become bound to sporozoite surfaces, and the de novo synthesis of α-gal by sporozoites [11]. *Plasmodium berghei* sporozoites reside in the skin for a considerable length of time after being inoculated by a biting anopheline, and this can facilitate immunological damage initiated by antibodies to α-gal [13, 14].

Early studies had suggested the presence of α-gal in cultured asexual blood stages of the human malaria parasite *P. falciparum* based on sensitivity to α-galactosidase treatment [15, 16], binding of *Bandeiraea simplicifolia* lectin isoform B4 [16, 17], and weak complement-dependent inhibition of asexual blood stage growth by natural antibodies to α-gal [7]. However, subsequent experiments showed that these were probably due to non-specific binding of the *Bandeiraea simplicifolia* lectin isoform B4 to *P. falciparum* lipids and the presence of cross-reactive oligosaccharide epitopes in *P. falciparum*-infected red blood cells [18]. *Plasmodium falciparum* lacks genes for many glycosyl transferases involved in synthesising *N*-glycans, glycophosphatidylinositol anchors and *O*-glycans compared to other eukaryotes [19, 20]. In vitro cultures of *P. falciparum* asexual blood stages did not incorporate galactose from UDP-galactose into glycolipids and glycoproteins when compared with parallel cultures of *Trypanosoma brucei* promastigotes [18]. BLASTp searches of the *P. falciparum* genome (GeneDB, Wellcome-Sanger Institute) with mammalian and other galactosyl transferases including a digalactosyl diglyceride synthase from *Arabidopsis thaliana* did not identify homologous proteins [18]. However, a *Staphylococcus aureus* galactosyl transferase with a function in synthesising cell wall components was significantly homologous to a *P. falciparum* *N*-acetylglucosaminyl phosphatidyl inositol synthase, an enzyme with a role in synthesising glycophosphatidylinositol membrane anchors [18], showing that BLASTp searches can reveal homologies in evolutionarily widely divergent organisms. These reports in conjunction with the present findings suggest that *Plasmodium* lacks the genetic ability to synthesise α-gal-containing glycoconjugates. This is consistent with the strong immune selection likely against malaria parasites expressing α-gal in catarrhines possessing natural antibodies to α-gal [21]. On the other hand, minimal or no immune selection against the expression of α-gal in vector mosquitoes is expected because their interaction with catarrhines only involves blood feeding.

The alternative of a mosquito origin for the α-gal detected on *P. berghei* and *P. falciparum* sporozoites [11] is therefore a likely explanation, as also proposed elsewhere [21, 22]. In migrating to salivary ducts from the haemocoele, *Plasmodium* sporozoites traverse a basement membrane and then move through salivary gland epithelial cells with many close intermolecular interactions occurring between the sporozoites and mosquito cells [23]. Mosquito-derived glycoconjugates can be transferred to the sporozoite membrane during these processes. This interpretation is consistent with the present finding of several putative GTs homologous to *I. scapularis* α1-3 GTs in *An. gambiae* and *Ae. aegypti*. It is also supported by demonstration of the transfer of labelled synthetic galactosyl glycoconjugates from *Anopheles stephensi* to *P. berghei* sporozoites in salivary glands [22], and the synthesis of α-gal in cultured cells derived from another important arboviral vector *Aedes albopictus* [24]. The presence of multiple proteins in *An. gambiae* and *Ae. aegypti* homologous to the three *I. scapularis* enzymes with α1-3 GT activity also suggests that the numbers of α and β GTs in mosquitoes may be increased as in *I. scapularis*, and this warrants further investigation.

The presence of α1-3 GT activity in ticks and mosquitos and the possible inoculation of α-gal-containing glycoconjugates into humans during blood feeding by the two types of arthropod vectors raise important issues regarding hypersensitivity reactions to their bites: (i) In the case of AGS hypersensitivity induced by tick vectors, it is not clear how IgE antibodies to α-gal are produced in the face of overwhelming concentrations of IgG and IgM antibodies to α-gal that might be expected to bind and remove relevant tick glycoconjugates from the inoculation site and blood. To explain this, it has been proposed that components such as prostaglandin E2 introduced with tick saliva during biting induce a T_H_2 bias and immunoglobulin class switching that favour the formation of IgE antibodies to α-gal [25]. It may also be relevant in this context that ticks are relatively rare human blood feeders. (ii) Mosquitos differ from ticks in that blood feeding is typically of a shorter duration, and bites are more frequent and may occur over many years in some locations. In comparison to tick bites, IgE and IgG antibodies may therefore have different temporal roles in the development of hypersensitivity to mosquito bites and an eventual desensitisation [1]. (iii) Little is presently known about the possible roles of α-gal or other carbohydrate epitopes in salivary glycoconjugates in causing hypersensitivity to mosquito bites. This gap in knowledge needs to be addressed.

Allergen immunotherapy with natural and recombinant salivary gland proteins is being developed for treating both AGS [4] and hypersensitivity caused by mosquito bites [1]. The possible effects of α-gal that can be present in the immunogens used for immunotherapy, however, requires careful consideration.

The four common human malaria parasite species, all of which had origins in African apes, have caused major genetic changes in humans associated with resistance to malaria [26]. It has been speculated that a selective advantage conferred by resistance to a pathogen [9], possibly an ancestral malaria parasite [7, 27], led to the inactivation of α1-3 GTs in catarrhines. The present data raise the possibility that neutralising the infective stages of pathogens, particularly ancestral malaria parasites, carrying vector-derived α-gal, and amelioration of hypersensitivity reactions may have been selective forces favouring the ability to make IgG and IgM antibodies to α-gal through the inactivation of α1-3 GT in catarrhines. The use of a variety of immunogens possessing terminal α-galactosyl residues to vaccinate against multiple human parasites (e.g. *Plasmodium* species, *Leishmania* species and *Trypanosoma cruzi*), human bacterial pathogens (e.g. *Borrelia burgdorferi*, *Anaplasma phagocytophilum* and *Mycobacterium* species) and veterinary pathogens (e.g. *Aspergillus fumigatus*, Newcastle disease virus) [28], as well as enveloped zoonotic viruses that infect humans (e.g. SARS-CoV-2) [29], has been proposed. The high concentration of natural antibodies to α-gal in blood and hypersensitivity reactions are factors that have to be carefully considered in this respect.

Because of likely similar evolution among arthropods, the mosquito proteins identified here are good candidates for enzymes involved in synthesising the α-gal that has been reported to be present in mosquito cells [11, 24]. Experimental verification of the synthesis of α-gal in mosquito salivary glands by the putative identified enzymes, and its transfer to *Plasmodium* sporozoites, is now needed to further understand primate evolution, hypersensitivity to mosquito bites and protection against malaria and other mosquito-borne pathogens.

## Supplementary Information

**Additional file 1.** Phylogenetic distance trees constructed with the NCBI BLASTp results.

## Data Availability

All data generated or analysed during this study are included in this published article and its Additional file 1.

## Abbreviations

α-gal: Galα1-3Galβ1-4GlcNAc-R
AGS: α-Gal syndrome
BLASTp: Basic Local Alignment Search Tool for proteins
Gal: Galactose
Gb: Globoside
GlcNAc: *N*-Acetyl glucosamine
GT: Galactosyl transferase
NCBI: National Center for Biotechnology Information
SARS-CoV-2: Severe acute respiratory syndrome coronavirus 2
T_H_2: T helper type 2 lymphocyte
