# Supplementary material for: Mosquito vector proteins homologous to α1-3 galactosyl transferases of tick vectors in the context of protective immunity against malaria and hypersensitivity to vector bites
Source: Parasit Vectors. 2021 Jun 5;14:303. doi: 10.1186/s13071-021-04801-7 (PMC8179703; doi:10.1186/s13071-021-04801-7)

**Additional file 1**. Phylogenetic distance trees constructed with the NCBI BLASTp results from the three *Ixodes scapularis* enzymes with α1-3 galactosyl transferase activity annotated as two α1-4 N-acetylglucosaminyl transferases (B7QKR3 and B7PLD1 in UniProt) and one annotated as a xylosylprotein β4-galactosyltransferase (B7PFJ6 in UniProt) and the unique homologous proteins from *Aedes aegypti* and *Anopheles gambiae* shown in Table 1. The *An. gambiae* proteins identified in the BLASTp analysis are shown with their VectorBase protein IDs. The *Ix. scapularis* query proteins are highlighted in yellow.


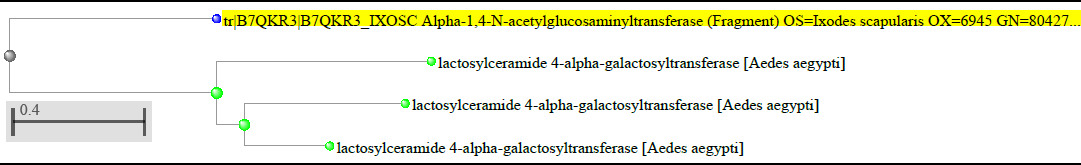


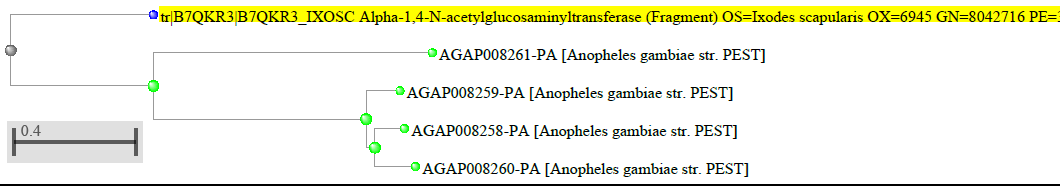


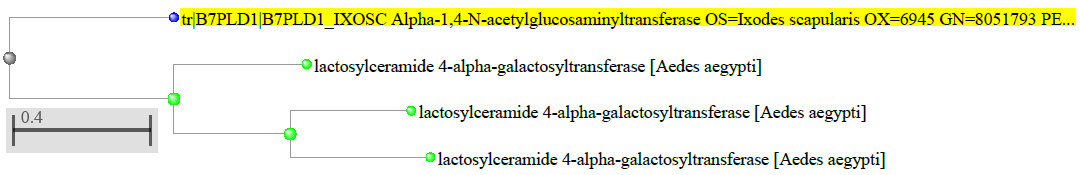


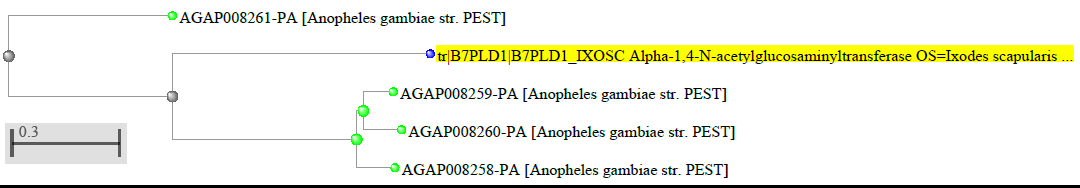


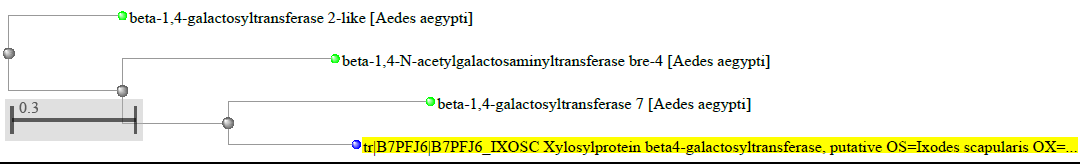


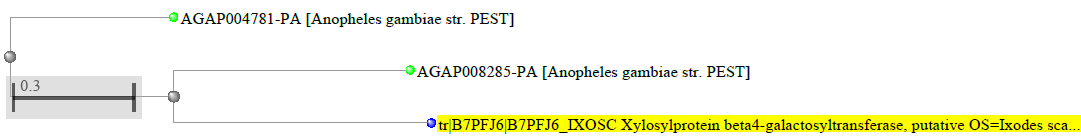

Supplement: Supplementary file 1 — Additional file 1. Phylogenetic distance trees constructed with the NCBI BLASTp results. [file 13071_2021_4801_MOESM1_ESM.docx]
